# Reconstructing Tone Sequences from Functional Magnetic Resonance Imaging Blood-Oxygen Level Dependent Responses within Human Primary Auditory Cortex

**DOI:** 10.3389/fpsyg.2017.01983

**Published:** 2017-11-14

**Authors:** Kelly H. Chang, Jessica M. Thomas, Geoffrey M. Boynton, Ione Fine

**Affiliations:** Department of Psychology, University of Washington, Seattle, WA, United States

**Keywords:** auditory, decoding, population receptive field, tonotopy, primary auditory cortex

## Abstract

Here we show that, using functional magnetic resonance imaging (fMRI) blood-oxygen level dependent (BOLD) responses in human primary auditory cortex, it is possible to reconstruct the sequence of tones that a person has been listening to over time. First, we characterized the tonotopic organization of each subject’s auditory cortex by measuring auditory responses to randomized pure tone stimuli and modeling the frequency tuning of each fMRI voxel as a Gaussian in log frequency space. Then, we tested our model by examining its ability to work in reverse. Auditory responses were re-collected in the same subjects, except this time they listened to sequences of frequencies taken from simple songs (e.g., “Somewhere Over the Rainbow”). By finding the frequency that minimized the difference between the model’s prediction of BOLD responses and actual BOLD responses, we were able to reconstruct tone sequences, with mean frequency estimation errors of half an octave or less, and little evidence of systematic biases.

## Introduction

A variety of blood-oxygen level dependent (BOLD) imaging studies have identified a pair of mirror-symmetric tonotopic gradients centered on Heschl’s gyrus on the cortical surface, thought to be the human homologs of primary areas A1 and R (Formisano et al., 2003; Woods et al., 2009; Humphries et al., 2010; Da Costa et al., 2011; Moerel et al., 2012; Saenz and Langers, 2014). These maps have been replicated across diverse imaging paradigms (Langers et al., 2014b; Da Costa et al., 2015; Thomas et al., 2015; Moerel et al., 2017) and a range of stimulus types including orderly frequency progressions (Talavage et al., 2004; Da Costa et al., 2011; Striem-Amit et al., 2011; Langers et al., 2014a), random tone sequences (Thomas et al., 2015), and complex natural stimuli (Moerel et al., 2012).

However, while the overall pattern of frequency gradients is highly replicable, the accuracy with which these maps have modeled the actual frequency preferences of individual voxels is unclear. For example, several groups (Formisano et al., 2003; Woods et al., 2009; Humphries et al., 2010; Langers et al., 2014a) have obtained robust tonotopic maps by evaluating BOLD responses to only a few discrete frequencies using a general linear model (GLM). However, these models fail to capture the explicit representation of frequency selectivity in the auditory cortex, which is thought to represent a wide range of auditory frequencies. Stimulus-specific biases can also alter the frequency preference assigned to a given fMRI voxel. Frequency “sweep” stimuli have been shown to induce a “traveling wave” of BOLD activity across the cortex (Engel et al., 1994) that is susceptible to biases induced by habituation and/or expectation effects as well as spatio-temporal BOLD non-linearities (Binda et al., 2013; Thomas et al., 2015). The complex morphology and small size of auditory cortical areas makes them highly susceptible to these biases (Saenz and Langers, 2014). Consequently, while the general topographic organization of PAC seems to be robust to the stimulus that was used, the frequency assigned to a given voxel can vary dramatically depending on the stimulus, for example the direction of the frequency sweep that is used (Da Costa et al., 2011; Thomas et al., 2015).

More recently, somewhat more complex modeling approaches have been applied to characterizing the response selectivities of auditory areas. One influential class of models has utilized an approach whereby natural scene stimuli are parameterized into a feature space and regularized linear regression is used to characterize each voxels response preference across this feature space (Kay et al., 2008; Naselaris et al., 2011; Nishimoto et al., 2011). The advantage of this approach is that it attempts to capture the complexity of cortical processing without explicitly imposing a preselected model (e.g., Gaussian tuning) upon the response selectivity profile for a given voxel (although the parameterization of the stimulus space must be appropriate). Voxel selectivity can be estimated as a weighted sum of the features to which the voxel responds. Recent papers using this approach have shown selectivity for, and interactions between, frequency, time, and spectro-temporal modulation (Santoro et al., 2014; Moerel et al., 2017).

The second class of models – the population receptive field (pRF) approach – has been equally influential. For this class, the response of the voxel is assumed to have a specific parameterized form (e.g., Gaussian tuning with log frequency) rather than allowing the stimulus to determine the selectivity profile. This provides an explicit function of voxel selectivity along the dimension(s) of interest (Dumoulin and Wandell, 2008; Zuiderbaan et al., 2012). Models of this class have tended to rely on relatively minimalist parameterizations (e.g., two parameters for a Gaussian in frequency space). Indeed, the popularity of this approach has rested in large part on its simplicity. One advantage is that it provides a clear test of how well a specific parameterized model of individual voxel tuning properties can predict BOLD responses within a given area. As a result, estimated parameter values can easily be compared across a wide range of stimulus paradigms, cortical areas, and subject groups.

Previously, we applied the pRF approach to auditory cortex to measure the frequency selectivity for individual voxels (Thomas et al., 2015). Here, we present a method for examining whether our simple model of frequency tuning can predict responses to more natural, familiar, and predictable stimuli. Specifically, we examined whether tonotopic maps generated using randomized tones could be used to decode and reconstruct a sequence of tones on the basis of an individual subjects’ BOLD responses over time. First, we characterized the tonotopic organization of each subject’s auditory cortex by measuring auditory responses to randomized pure tone stimuli and modeling the frequency tuning of each fMRI voxel as a Gaussian in log frequency space. Next, we measured cortical responses in the same subjects to novel stimuli containing a sequence of tones based on the melodies “When You Wish Upon a Star” (Harline et al., 1940) and “Over the Rainbow” (Arlen and Harburg, 1939). These ‘song-like’ sequences were chosen because they include complex temporal dependencies as well as expectation effects, albeit over a very slow time scale. Then, using a parametric decoding method, we reconstructed the tones from these songs by determining what frequency would best maximize the correlation between predicted (based on our pRF models) and obtained BOLD activity patterns for each point in the stimulus time course.

## Materials and Methods

Three right-handed subjects (2 male, 1 female, ages 27–46) participated in two fMRI sessions. Subjects reported normal hearing and no history of neurological or psychiatric illness. Written informed consent was obtained from all subjects and procedures, including recruitment and testing, followed the guidelines of the University of Washington Human Subjects Division and were reviewed and approved by the Institutional Review Board.

### MRI Data Acquisition and Analysis

Blood-oxygen level dependent imaging was performed using a 3 Tesla Philips Achieva scanner (Philips, Eindhoven, The Netherlands) at the University of Washington Diagnostic Imaging Sciences Center (DISC). Subjects were instructed to keep their eyes closed throughout all scans and foam padding was used to minimize head motion. fMRI data were acquired using a 32-channel head coil and a continuous EPI pulse sequence (2.8 mm × 2.8 mm × 2.8mm, TR/TE = 2000/25 ms, flip angle = 60°, EPI-factor = 35, no slice gap). We used a continuous sequence designed with Philips SofTone software (SofTone factor of 4.0) to generate less acoustic scanner noise (Thomas et al., 2015).

Standard pre-processing of fMRI data was carried out using BrainVoyager QX software (version 2.3.1, Brain Innovation B.V., Maastricht, The Netherlands), including 3D motion correction, slice scan time correction, and temporal high-pass filtering. 3D motion correction was performed by aligning to all volumes to the first volume within a session on 9 parameters for translation, rotation, and scale. Slice scan time correction was performed using cubic spline interpolation with an ascending and interleaved order of the slice scan acquisition. Temporal high-pass filtering was performed at a cutoff of 2 cycles per scan. Functional data were aligned to a T1-weighted anatomical image acquired in the same session (MPRAGE, 1 mm × 1 mm × 1mm). The anatomical images acquired in the two sessions were aligned to each other and to each subject’s 3D Talairach-normalized functional dataset. The BrainVoyager QX automatic segmentation routine was used to reconstruct the cortical surface and the resulting smooth 3D surface was partially inflated. For each subject, large anatomical regions of interest (ROIs) were selected from both hemispheres of the auditory cortical surface using drawing tools within BrainVoyager QX. Preprocessed time-course data for each 3D anatomical voxel within the volume ROI were then exported to MATLAB for further analysis.

### Auditory Stimulus Presentation

Sound stimuli were generated in MATLAB using the Psychophysics Toolbox^1^. Stimuli were delivered via MRI compatible insert earphones (S14, Sensimetrics), at a sampling rate of 44.1 kHz, with intensities calibrated to ensure flat frequency transmission from 100 to 8 kHz. After sound system calibration, stimulus sound intensities were adjusted according to a standard equal-loudness curve created for insert earphones (ISO 226: 2003) to approximate equal perceived loudness across all frequencies. Acoustic noise from the scanner was attenuated by expanding-foam ear tips as well as protective ear muffs placed over the ear following earphone insertion. Subjects reported hearing all stimuli at a clear and comfortable audible level, with roughly equal loudness across all tones.

### pRF Estimation

**FIGURE 1:**
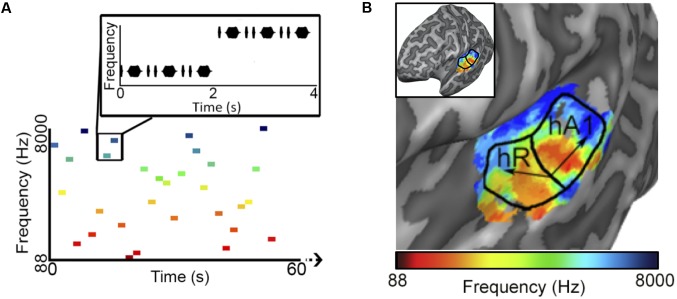
Population receptive field (pRF) estimation. **(A)** The first 60 s of an example *random sequence* stimulus used during pRF estimation. Each block lasted 2 s and consisted of 8 pure tone bursts of a single frequency. Bursts lasted either 50 or 200 ms in duration (inter-stimulus interval = 50 ms) and were presented in a pseudo-randomized order. **(B)** Tonotopic and bandwidth maps for the left hemisphere of example Subject 1. As indicated by the black arrows, pRF frequency center (Hz) values formed two mirror-symmetric tonotopic gradients corresponding to the primary auditory fields A1 and R, outlined here by solid black lines. No clear organization was observed for pRF bandwidth (octaves) values (not shown).

To reduce the influence of spatiotemporal non-linearities on pRF estimates, we measured fMRI responses to randomized pure tone sequences consisting of 240 frequency blocks. As shown in **Figure 1A**, each block lasted 2 s and consisted 8 pure tone bursts of a single frequency. Each burst lasted either 50 or 200 ms in duration (inter-stimulus interval = 50 ms) and was presented in a pseudo-randomized order, resulting in a “Morse code” like pattern of tones. This served to increase the perceptual salience of the tone bursts over the background scanner noise. The frequencies presented in the blocks were equally spaced on a logarithmic scale, ranging from 88 to 8000 Hz. Each frequency block was presented only once per scan and block order was randomly shuffled for each scan. Following every 60 blocks was a 12 s silent pause. This silent period allows the pRF algorithm to better estimate the baseline fMRI response to scanner noise. Each subject participated in a single pRF estimation scanning session, consisting of 6 scans, each containing a different randomized sequence of the same 240 frequency blocks.

Following previously described methods, we used customized MATLAB software to estimate the frequency tuning curves for individual voxels based on a linear temporal model of the fMRI BOLD response time course (Thomas et al., 2015). Briefly, analysis began by defining a stimulus time course, which indicates the presence or absence of a particular frequency over time. This stimulus time course was convolved with each subject’s estimated hemodynamic response function (HDR) modeled as a gamma probability density function (Boynton et al., 1996). Each voxel’s response was modeled using a 1-dimensional Gaussian function g*(f)*, defined over frequency (in log space). The center (f_0_) of each Gaussian corresponds to the frequency of the voxel’s maximum sensitivity, and the standard deviation (σ) corresponds to the range of frequencies that the voxel is sensitive to. Standard deviations are reported as bandwidth values by calculating the full width half maximum (FWHM) in terms of octaves. A predicted fMRI time course was then generated for each voxel by calculating the linear sum of the overlap between the hemodynamically blurred stimulus time course and the pRF model. Finally, model fits for each voxel were obtained using a non-linear search algorithm that found the model parameters that maximized the correlation value (goodness-of-fit) between the voxel’s pRF predicted time course and the acquired fMRI BOLD response time course (using Matlab’s “fmincon” function).

The procedure described above included a few modifications from our original pRF analysis (Thomas et al., 2015). First, we included a static power-law non-linearity within the Gaussian model by including a free exponent parameter (*n*) to account for non-linear summation of the BOLD response according to the compressive spatial summation (CSS) model (Kay et al., 2013). The incorporation of this static non-linearity, which is applied after the initial fitting of the linear model, has been shown to more accurately explain BOLD activity and improve overall receptive field fits. This parameter was constrained to fall between 0 and 1. Second, we constrained the Gaussian standard deviation (σ) to values greater than 0.015 (chosen based on the resolution of the presented frequencies).

The drawing of the PAC region of interest was performed using both the functional data and following anatomical landmarks. A second independent observer verified the selection of the ROI.

After fitting, only voxels within PAC, with a pRF correlation value (goodness-of-fit) above 0.15 were retained for song decoding and reconstruction (533, 530, 244 voxels for subjects S1–S3, respectively). Results were robust to a wide range of correlation values. Critically, *all* voxels with pRF fits above this threshold within PAC were included in all further analyses, there was no further selection based on the ability to predict the song-like stimuli. Thus, there was no selection of voxels on the basis of their ability to generalize to a novel stimulus. As demonstrated in **Figure 1B**, pRF center (f_0_) values formed two mirror-symmetric tonotopic gradients corresponding to the primary auditory fields A1 and R in both hemispheres of all subjects. No clear topographical organization within PAC was observed for either pRF bandwidth values (average bandwidth in octaves ± *SD*, S1 = 3.385 ± 2.807, S2 = 3.732 ± 1.634, S3 = 2.219 ± 1.201), or exponent parameters (average value of *n* ± *SD*; S1 = 0.587 ± 0.310, S2 = 0.611 ± 0.228, and S3 = 0.726 ± 0.318).

### Frequency Decoding

During a separate scanning session, we collected fMRI responses to two pure tone song-like sequences based on two familiar melodies: “When You Wish Upon a Star” (Wish) and “Somewhere Over the Rainbow” (Rainbow). Each song-like sequence was generated using 2 s frequency blocks with frequencies ranging from 880 to 2349 Hz (corresponding to the notes A5-D7 on the western music scale). Each 2 s block contained 13 tone bursts of the same frequency, each lasting 75 ms in duration (inter-stimulus interval = 75 ms). This created a vibrato-like effect which served to increase the perceptual salience of each block, without interrupting the melodic feel of the song-like sequence. A single presentation of each song-like sequence contained either 25 (Wish) or 23 (Rainbow) frequency blocks followed by 8 s of silence, and the entire presentation was repeated 8 times per scan. Averaged fMRI BOLD time courses were then generated for each song-like sequence by averaging data responses across the eight presentations within each scan, and across two scans of the same sequence type.

We decoded both song-like sequences by reconstructing each sequence one block at a time. To do this, we used the pRF models previously generated with the randomized tone sequences to generate predicted voxel activity patterns elicited for a set of 14 frequencies sampled from 88 to 8000 Hz in half-octave steps. The best fitting frequency from this set is then used as the initial parameter for a non-linear optimization fitting procedure (again, Matlab’s “fmincon” function) that determined what frequency produced the predicted voxel activity pattern best correlated with the measured voxel activity pattern for each 2 s block. This process was then repeated for each block in the sequence, until all frequency blocks had been reconstructed. Finally, to account for the delayed hemodynamic blurring of BOLD signal a fixed temporal lag of 6 s was applied to the reconstructed sequence (Kay et al., 2008).

It is important to note that our method only depends on the frequency selectivity of individual voxels, not their physical locations within auditory cortex. This method is therefore not dependent upon any particular model (Saenz and Langers, 2014; Moerel et al., 2015) of frequency selectivity organization.

The quality of the reconstructed sequences was quantified in three ways: *Identification performance*, *reconstruction accuracy, and model reliability*.

*Identification performance* was assessed as the ability to correctly identify the actual song over other song-like sequences that contained similar statistical properties. For each reconstructed sequence, we applied an algorithm based on first-order Markov chains to randomly generate 1000 simulated (new sequences were generated for each subject) song-like sequences that reflected the frequency content and note-to-note probabilities of the Rainbow and Wish sequences. Other more advanced methods for generating simulated sequences exist, including probabilistic models of melodic intervals (Temperley, 2008, 2014). However, our model was generated using unpredictable stimuli, and did not incorporate any information about interval dependencies. Consequently, identification performance was unlikely to be significantly altered by the use of more realistic foil sequences. We then calculated the correlation (Pearson’s *r*) between the reconstructed sequence and the actual sequence of tones, as well as for each of the simulated foils. Identification performance was defined as the number of times in which the actual sequence was correctly selected, on the basis of having a higher correlation with the reconstructed sequence than any of the 1000 simulated sequences.

*Reconstruction accuracy* was assessed as the ability to recreate each note in the actual sequence. This was calculated as the residual difference in cents (1200 cents per octave) between each note in the reconstructed and actual sequences. To determine if any systematic over or underestimation was present in the reconstructed sequences, we performed a two-tailed *t*-test on the means of the residual errors. Any mean that was significantly different from zero reflected an overall bias in reconstruction accuracy.

*Model reliability* was assessed using the metric of relative root mean square error (rRMSE, Rokem et al., 2015). For both song-sequences we normalized the root mean square error (RMSE) value describing the difference between predicted and measured time series by the RMSE describing the difference between the time series of each of the two scans collected for that song-sequence. Thus, for each song:

$$rRMSE=\frac{\left(RMSE({TC}_{pred},{TC}_{scan\,\;1}\,)+RMSE\left({TC}_{pred},{TC}_{scan\,\;2}\right)\right)}{2RMSE\left({TC}_{scan\,\;1},{TC}_{scan\,\;2}\right)}$$

Where TC_scan 1_, and TC_scan 2_ are the measured time course for the song-like sequence obtained in individual scans. TC_pred_ is the predicted time course for the song-like sequence, based on our pRF model (generated using random tones). This measure provides us with an index of the goodness-of-fit of our model, relative to measurement reliability. As described by Rokem et al. (2015), if the model has higher accuracy than test–retest accuracy then rRMSE < 1. For simple cases of IID signals with zero-mean Gaussian noise, if the model perfectly captured the data then $rRMSE=\frac{1}{\sqrt{2}}=0.707$.

## Results

**FIGURE 2:**
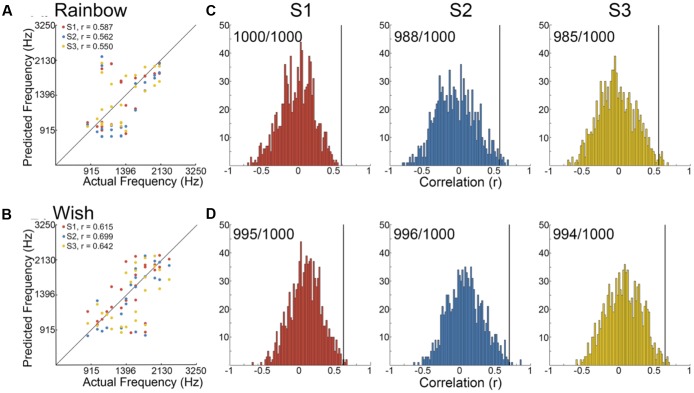
Identification performance. **(A,B)** Scatter plots showing the correlation between reconstructed and actual frequencies for each subject for both the Rainbow **(A)** and Wish **(B)** sequences, also shown **Table 1**. **(C,D)** Using a method based on a first order Markov chain algorithm, we simulated 1000 song-like sequences reflecting the frequency content and note-to-note probabilities of the Rainbow **(C)** and Wish **(D)** sequences. Histograms show the distribution of correlation values (Pearson’s *r*) between each of the simulated sequences and either reconstructed sequence. The line in black designates the correlation value between the actual song-like sequences and the reconstructed sequences, indicating the degree to which the correct sequence had been successfully identified. The number of correct identifications (out of 1000) is reported for each reconstructed sequence. Colors correspond to individual subjects.

We began by determining the correlation between reconstructed and actual frequencies for each subject for both Rainbow and Wish (**Figures 2A,B** and **Table 1**) sequences. For all subjects, reconstructed sequences were well correlated with the actual sequences that were presented, indicating good reconstruction accuracy.

**Table 1:** Model performance: Reconstruction accuracy.

Subject	Stimulus	Pearson’s *r*	Residual Errors (cents) Mean ± *SD*
**S1**	Rainbow	0.587	25.98 ± 465.44
	Wish	0.615	-30.71 ± 448.05
**S2**	Rainbow	0.562	-210.12 ± 512.17
	Wish	0.699	-215.54 ± 421.35
**S3**	Rainbow	0.550	14.6 ± 456.24
	Wish	0.642	-155.78 ± 434.02


**Correlation values (Pearson’s *r, also shown **Figure 2***) between reconstructed and actual frequency values, mean and standard deviations of residual errors in cents between the reconstructed and actual frequencies.**

**Figures 2C,D** illustrate identification performance. Histo grams containing the correlation between the reconstructed Rainbow (**Figure 2C**) and Wish (**Figure 2D**) sequences and 1000 simulated foils. The correlation value between the predicted and the actual sequence is represented by a black line in each histogram, indicating the correlation value for the actual sequence. Identification performance for both Rainbow and Wish was at near perfect levels for all three subjects, demonstrating that the identity of a tone sequence can be readily be decoded based on the similarity between the predicted BOLD response to that sequence of tones and the measured BOLD response.

**FIGURE 3:**
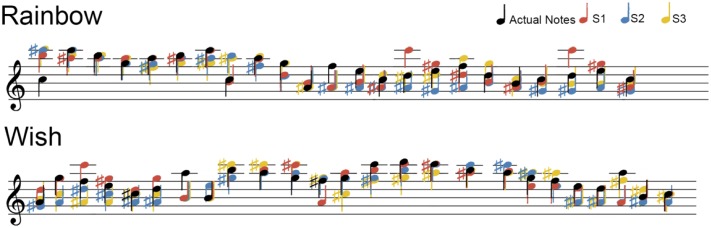
Sequence Reconstruction. For easier visualization on a treble clef, all frequencies (Hz) were rounded the nearest semitone and lowered one octave. Actual notes from each song-like sequence are in black, while the color of notes in the reconstructed sequences corresponds to individual subjects.

**Figure 3** displays the notes of the actual and reconstructed sequences of each subject on the five-line staff according to modern musical notation. Purely for illustration purposes, the reconstructed frequencies in **Figure 3** were rounded to the nearest semitone (12 semitones per octave), or “note.” We also lowered all notes (actual and reconstructed) one octave for better representation on the treble clef. One way of assessing the precision of our pRF decoding method is by examining how accurately each song-like sequence was reconstructed in terms of musical intervals or cents. The standard deviations of the residual errors are reported in **Table 1**. Standard deviations ranged between 434 and 512 cents across subjects and songs (around three to four notes, or a third of an octave).

We also examined whether the mean of the residual errors differed significantly from zero, which would reflect a systematic bias in reconstruction accuracy (**Table 1**). Of the six means, only one reached statistical significance with a two-tailed *t*-test [Subject 2, Wish, *t*(24) = -215.54 cents, *p* = 0.0173], non-significant after either Bonferroni or Bonferroni-Holm correction (Holm, 1979, 3). Thus, there does not appear to be a systematic over or underestimation of reconstructed frequencies, at least as far as the power of our experimental design can provide.

**FIGURE 4:**
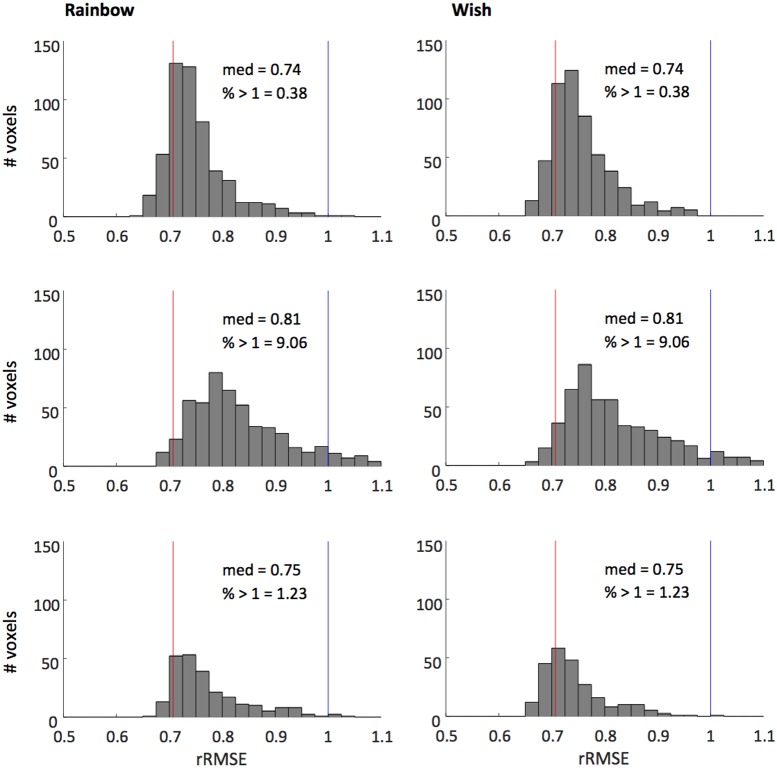
Histograms of voxel rRMSE values for 2 song-like sequences and 3 subjects. The population receptive field model predicts the data better than test–retest reliability (blue line, rRMSE = 1) in almost all voxels. Median rRMSE values are close to the expected performance of a perfect model (red line, rRMSE = 0.707). Inset text show median rRMSE and the percentage of voxels > 1.

**Figure 4** shows that our model fits the novel song-sequences extremely accurately. The blue line shows rRMSE = 1, representing performance equal to test–retest reliability. For all subjects and song-sequences most of the voxels had rRMSE values < 1. Indeed, for 2 of the 3 subjects fewer than 1% of voxels had rRMSE values greater than 1. The red line shows rRMSE = 0.707: the expected performance value if the model was perfect (assuming zero-mean IID noise). The median rRMSE values for all three subjects were close to the expected value of a perfect model, with only small room for improvement.

## Discussion

Using a combined auditory pRF encoding/decoding approach, we found that we could accurately identify and reconstruct tone sequences over time on the basis of BOLD responses, thereby demonstrating the predictive accuracy of our model of frequency selectivity of PAC.

### Encoding/Decoding Models of Sensory Cortex

A few previous studies have used linear classifier algorithms, trained to discriminate between stimulus categories based on patterns of activity across fMRI voxels, to classify speech content and speaker identity (Formisano et al., 2008) as well as the emotional content of speech (Ethofer et al., 2009). One limitation of such classification approaches is that they are limited to candidate stimulus sets and cannot be generalized to substantially novel stimuli (Naselaris et al., 2011). Moreover, linear classifiers do not provide insight into the feature space of functional organization within auditory cortex (see, Naselaris and Kay, 2015, for discussion).

Another fundamental difference between our study and the linear classification studies described above, is that linear classifiers select the components in the response state with the greatest predictive value. Critically, for both identification and reconstruction we used *all* voxels within PAC whose responses could be fit by the pRF model. Thus, identification performance did not assess whether *any* voxels in PAC could successfully identify the tone sequence that was presented, but rather assessed whether the collective responses of voxels within PAC *as a whole* carries reliable and generalizable information about the tone sequence.

As described in the Section “Introduction,” there currently exist two classes of models that are designed to carry out encoding/decoding that is generalizable to novel stimuli. The first relies on a parameterization of the *stimulus* space through single-voxel encoding (Kay et al., 2008; Naselaris et al., 2009; Nishimoto et al., 2011; Moerel et al., 2014; Santoro et al., 2014) and multivariable model-based approaches (Miyawaki et al., 2008; Santoro et al., 2017). Two previous studies (Moerel et al., 2017; Santoro et al., 2017) have used this approach to examine decoding and reconstruction performance for 1 s natural auditory scenes. In both studies responses to a training set were used to estimate each voxel’s sensitivity to a range of spectrotemporal features. Sensitivity was described on the basis of models of varying degrees of complexity, ranging from simple frequency to a 4D model that included frequency, spectral modulation, temporal modulation and time. In the Moerel et al. (2017) study the model was assessed by computing the correlation between the models predicted time course to a given sound and the measured time courses to the remainder of the test sounds. In the Santoro et al. (2017) study, voxel responses to a test set, in conjunction with the voxel weightings across the feature space, were used to reconstruct the features of each test stimulus.

Our model belongs to the second class of models – our goal was to specifically model the response selectivity of the voxel with an assumed Gaussian selectivity profile. Our stimuli and model only varied along the dimension of frequency, because we wanted to examine the pRF approach using a dimension whose representation within PAC is reasonably well characterized. Having shown that our model can identify what song-like sequence a person had been listening to with high reliability, we also demonstrate that a pRF model of tonotopic organization in the human primary auditory cortex can also *reconstruct* the sequence of tones played over time. Our *encoding* pRF model was used to describe the frequency selectivity of individual voxels in each subject’s primary auditory cortex (Thomas et al., 2015). Then we applied a parametric *decoding* method on our pRF model to identify and reconstruct tone sequences. We examined the reliability and validity of our tonotopic encoding model in a variety of quantitative ways. Identification performance was virtually perfect. Reconstruction accuracy of single tones was also excellent, we were able to reconstruct the tones of the song-like stimuli for all three subjects within a half of an octave or less, with little evidence of systematic biases in frequency estimation. Finally, and importantly, our rRMSE estimate of model accuracy suggests that our model, despite being much simpler than these other models, is nearly perfect: the model (generated using random tones) predicted the time course of song-sequences far better than test–retest reliability. Indeed, rRMSE estimates of model performance suggested that our model performed close to optimally, despite these novel stimuli containing a more restricted range of frequencies, greater temporal dependencies, and (presumably) expectation effects. This suggests that, for the stimuli used here, these factors did not radically alter the tonotopic information carried by individual voxels.

As described above, other studies have shown that neurons in auditory cortex respond selectively to other stimulus dimensions, including spectral and temporal modulation, time and loudness (Langner et al., 1997, 2009; Sadagopan and Wang, 2009; Schonwiesner and Zatorre, 2009; Baumann et al., 2011; Barton et al., 2012; Moerel et al., 2012, 2013, 2015, 2017; Santoro et al., 2014, 2017; Uppenkamp and Röhl, 2014). However, while recent studies (Moerel et al., 2017; Santoro et al., 2017) make it clear that voxels vary in their responsivity across these various dimensions, there is still much to be learned about how topographical selectivity for these other dimensions vary within primary and secondary auditory areas, and whether there are systematic differences in selectivity across these various dimensions across different cortical areas. Discovering parameterizations that can simplify this multidimensional space by summarizing voxel selectivity across multiple dimensions would be a natural extension of our approach. At some point it is likely that our approach (building up from simple stimuli and simple models) and that of other groups using more complex stimuli and models (Moerel et al., 2017; Santoro et al., 2017) will converge at an optimal level of model complexity.

One promising future direction will be inclusion of the effects of temporal regularities. The stimuli used to develop our pRF model did not contain any first or second order statistical regularities, and thus our model does not capture the effects of attention, expectation, or longer-term habituation (our model did include response compression) on the BOLD response, despite these factors being known to strongly modulate auditory cortex responses (Da Costa et al., 2013; Thomas et al., 2015). However, as described above, we were able to use pRFs based on responses to unpredictable stimuli to reconstruct the fMRI time courses to predictable song-like stimuli with nearly equal accuracy as for the unpredictable stimuli.

Other promising future directions include using a large number of subjects to examine variability in the population, using these methods to link cortical responses to perceptual experience for ambiguous auditory stimuli, examining whether cortical representations can predict behavioral performance in both typical and atypical populations, and examining the effects of frequency-selective attention (Woods et al., 2009; Da Costa et al., 2013).

## Author Contributions

All authors designed the research. KC and JT collated and analyzed the data. All authors drafted, provided critical revisions, and approved the final draft of the manuscript.

## Conflict of Interest Statement

The authors declare that the research was conducted in the absence of any commercial or financial relationships that could be construed as a potential conflict of interest.

## Footnotes

1 www.psychtoolbox.org

## References

[B1] Arlen H. Harburg E. Y. (1939). *Over the Rainbow : From the M-G-M Picture, the Wizard of Oz*. New York, NY: L. Feist.

[B2] Barton B. Venezia J. H. Saberi K. Hickok G. Brewer A. A. (2012). Orthogonal acoustic dimensions define auditory field maps in human cortex. *Proc. Natl. Acad. Sci. U.S.A.* 109 20738–20743. 10.1073/pnas.1213381109 23188798 PMC3528571

[B3] Baumann S. Griffiths T. D. Sun L. Petkov C. I. Thiele A. Rees A. (2011). Orthogonal representation of sound dimensions in the primate midbrain. *Nat. Neurosci.* 14 423–425. 10.1038/nn.2771 21378972 PMC3068195

[B4] Binda P. Thomas J. M. Boynton G. M. Fine I. (2013). Minimizing biases in estimating the reorganization of human visual areas with BOLD retinotopic mapping. *J. Vis.* 13 13. 10.1167/13.7.13 23788461 PMC3689563

[B5] Boynton G. M. Engel S. A. Glover G. H. Heeger D. J. (1996). Linear systems analysis of functional magnetic resonance imaging in human V1. *J. Neurosci.* 16 4207–4221. 8753882 10.1523/JNEUROSCI.16-13-04207.1996 PMC6579007

[B6] Da Costa S. Saenz M. Clarke S. Van Der Zwaag W. (2015). Tonotopic gradients in human primary auditory cortex: concurring evidence from high-resolution 7 T and 3 T fMRI. *Brain Topogr.* 28 66–69. 10.1007/s10548-014-0388-0 25098273

[B7] Da Costa S. Van Der Zwaag W. Marques J. P. Frackowiak R. S. Clarke S. Saenz M. (2011). Human primary auditory cortex follows the shape of Heschl’s gyrus. *J. Neurosci.* 31 14067–14075. 10.1523/JNEUROSCI.2000-11.2011 21976491 PMC6623669

[B8] Da Costa S. Van Der Zwaag W. Miller L. M. Clarke S. Saenz M. (2013). Tuning in to sound: frequency-selective attentional filter in human primary auditory cortex. *J. Neurosci.* 33 1858–1863. 10.1523/JNEUROSCI.4405-12.2013 23365225 PMC4340971

[B9] Dumoulin S. O. Wandell B. A. (2008). Population receptive field estimates in human visual cortex. *Neuroimage* 39 647–660. 10.1016/j.neuroimage.2007.09.034 17977024 PMC3073038

[B10] Engel S. A. Rumelhart D. E. Wandell B. A. Lee A. T. Glover G. H. Chichilnisky E. J. (1994). fMRI of human visual cortex. *Nature* 369 525. 10.1038/369525a0 8031403

[B11] Ethofer T. Van De Ville D. Scherer K. Vuilleumier P. (2009). Decoding of emotional information in voice-sensitive cortices. *Curr. Biol.* 19 1028–1033. 10.1016/j.cub.2009.04.054 19446457

[B12] Formisano E. De Martino F. Bonte M. Goebel R. (2008). “Who” is saying “what”? Brain-based decoding of human voice and speech. *Science* 322 970–973. 10.1126/science.1164318 18988858

[B13] Formisano E. Kim D. S. Di Salle F. Van De Moortele P. F. Ugurbil K. Goebel R. (2003). Mirror-symmetric tonotopic maps in human primary auditory cortex. *Neuron* 40 859–869. 10.1016/S0896-6273(03)00669-X 14622588

[B14] Harline L. Washington N. Disney W. Edwards C. Young V. Victor Young O. (1940). *When you Wish Upon a Star (from Walt Disney’s “Pinocchio”)*, Chap. Dubuque, IA. Decca.

[B15] Holm S. (1979). A simple sequentially rejective multiple test procedure. *Scand. J. Stat.* 6 65–70.

[B16] Humphries C. Liebenthal E. Binder J. R. (2010). Tonotopic organization of human auditory cortex. *Neuroimage* 50 1202–1211. 10.1016/j.neuroimage.2010.01.046 20096790 PMC2830355

[B17] Kay K. N. Naselaris T. Prenger R. J. Gallant J. L. (2008). Identifying natural images from human brain activity. *Nature* 452 352–355. 10.1038/nature06713 18322462 PMC3556484

[B18] Kay K. N. Winawer J. Mezer A. Wandell B. A. (2013). Compressive spatial summation in human visual cortex. *J. Neurophysiol.* 110 481–494. 10.1152/jn.00105.2013 23615546 PMC3727075

[B19] Langers D. R. Krumbholz K. Bowtell R. W. Hall D. A. (2014a). Neuroimaging paradigms for tonotopic mapping (I): the influence of sound stimulus type. *Neuroimage* 100 650–662. 10.1016/j.neuroimage.2014.07.044 25069046 PMC5548253

[B20] Langers D. R. Sanchez-Panchuelo R. M. Francis S. T. Krumbholz K. Hall D. A. (2014b). Neuroimaging paradigms for tonotopic mapping (II): the influence of acquisition protocol. *Neuroimage* 100 663–675. 10.1016/j.neuroimage.2014.07.042 25067814 PMC5546393

[B21] Langner G. Dinse H. R. Godde B. (2009). A map of periodicity orthogonal to frequency representation in the cat auditory cortex. *Front. Integr. Neurosci.* 3:27. 10.3389/neuro.07.027.2009 19949464 PMC2784045

[B22] Langner G. Sams M. Heil P. Schulze H. (1997). Frequency and periodicity are represented in orthogonal maps in the human auditory cortex: evidence from magnetoencephalography. *J. Comp. Physiol. A* 181 665–676. 10.1007/s003590050148 9449825

[B23] Miyawaki Y. Uchida H. Yamashita O. Sato M. A. Morito Y. Tanabe H. C. (2008). Visual image reconstruction from human brain activity using a combination of multiscale local image decoders. *Neuron* 60 915–929. 10.1016/j.neuron.2008.11.004 19081384

[B24] Moerel M. De Martino F. Formisano E. (2012). Processing of natural sounds in human auditory cortex: tonotopy, spectral tuning, and relation to voice sensitivity. *J. Neurosci.* 32 14205–14216. 10.1523/JNEUROSCI.1388-12.2012 23055490 PMC6622378

[B25] Moerel M. De Martino F. Formisano E. (2014). An anatomical and functional topography of human auditory cortical areas. *Front. Neurosci.* 8:225 10.3389/fnins.2014.00225 PMC4114190 25120426

[B26] Moerel M. De Martino F. Kemper V. G. Schmitter S. Vu A. T. Ugurbil K. (2017). Sensitivity and specificity considerations for fMRI encoding, decoding, and mapping of auditory cortex at ultra-high field. *Neuroimage* 10.1016/j.neuroimage.2017.03.063 [Epub ahead of print]. 28373123 PMC5623610

[B27] Moerel M. De Martino F. Santoro R. Ugurbil K. Goebel R. Yacoub E. (2013). Processing of natural sounds: characterization of multipeak spectral tuning in human auditory cortex. *J. Neurosci.* 33 11888–11898. 10.1523/JNEUROSCI.5306-12.2013 23864678 PMC3713728

[B28] Moerel M. De Martino F. Santoro R. Yacoub E. Formisano E. (2015). Representation of pitch chroma by multi-peak spectral tuning in human auditory cortex. *Neuroimage* 106 161–169. 10.1016/j.neuroimage.2014.11.044 25479020 PMC4388253

[B29] Naselaris T. Kay K. N. (2015). Resolving ambiguities of MVPA using explicit models of representation. *Trends Cogn. Sci.* 19 551–554. 10.1016/j.tics.2015.07.005 26412094 PMC4748837

[B30] Naselaris T. Kay K. N. Nishimoto S. Gallant J. L. (2011). Encoding and decoding in fMRI. *Neuroimage* 56 400–410. 10.1016/j.neuroimage.2010.07.073 20691790 PMC3037423

[B31] Naselaris T. Prenger R. J. Kay K. N. Oliver M. Gallant J. L. (2009). Bayesian reconstruction of natural images from human brain activity. *Neuron* 63 902–915. 10.1016/j.neuron.2009.09.006 19778517 PMC5553889

[B32] Nishimoto S. Vu A. T. Naselaris T. Benjamini Y. Yu B. Gallant J. L. (2011). Reconstructing visual experiences from brain activity evoked by natural movies. *Curr. Biol.* 21 1641–1646. 10.1016/j.cub.2011.08.031 21945275 PMC3326357

[B33] Rokem A. Yeatman J. D. Pestilli F. Kay K. N. Mezer A. Van Der Walt S. (2015). Evaluating the accuracy of diffusion MRI models in white matter. *PLOS ONE* 10:e0123272. 10.1371/journal.pone.0123272 25879933 PMC4400066

[B34] Sadagopan S. Wang X. (2009). Nonlinear spectrotemporal interactions underlying selectivity for complex sounds in auditory cortex. *J. Neurosci.* 29 11192–11202. 10.1523/JNEUROSCI.1286-09.2009 19741126 PMC2757444

[B35] Saenz M. Langers D. R. (2014). Tonotopic mapping of human auditory cortex. *Hear. Res.* 307 42–52. 10.1016/j.heares.2013.07.016 23916753

[B36] Santoro R. Moerel M. De Martino F. Goebel R. Ugurbil K. Yacoub E. (2014). Encoding of natural sounds at multiple spectral and temporal resolutions in the human auditory cortex. *PLOS Comput. Biol.* 10:e1003412. 10.1371/journal.pcbi.1003412 24391486 PMC3879146

[B37] Santoro R. Moerel M. De Martino F. Valente G. Ugurbil K. Yacoub E. (2017). Reconstructing the spectrotemporal modulations of real-life sounds from fMRI response patterns. *Proc. Natl. Acad. Sci. U.S.A.* 114 4799–4804. 10.1073/pnas.1617622114 28420788 PMC5422795

[B38] Schonwiesner M. Zatorre R. J. (2009). Spectro-temporal modulation transfer function of single voxels in the human auditory cortex measured with high-resolution fMRI. *Proc. Natl. Acad. Sci. U.S.A.* 106 14611–14616. 10.1073/pnas.0907682106 19667199 PMC2732853

[B39] Striem-Amit E. Hertz U. Amedi A. (2011). Extensive cochleotopic mapping of human auditory cortical fields obtained with phase-encoding FMRI. *PLOS ONE* 6:e17832. 10.1371/journal.pone.0017832 21448274 PMC3063163

[B40] Talavage T. M. Sereno M. I. Melcher J. R. Ledden P. J. Rosen B. R. Dale A. M. (2004). Tonotopic organization in human auditory cortex revealed by progressions of frequency sensitivity. *J. Neurophysiol.* 91 1282–1296. 10.1152/jn.01125.2002 14614108

[B41] Temperley D. (2008). A probabilistic model of melody perception. *Cogn. Sci.* 32 418–444. 10.1080/03640210701864089 21635341

[B42] Temperley D. (2014). Probabilistic models of melodic interval. *Music Percept.* 32 85–99. 10.1525/mp.2014.32.1.85

[B43] Thomas J. M. Huber E. Stecker G. C. Boynton G. M. Saenz M. Fine I. (2015). Population receptive field estimates of human auditory cortex. *Neuroimage* 105 428–439. 10.1016/j.neuroimage.2014.10.060 25449742 PMC4262557

[B44] Uppenkamp S. Röhl M. (2014). Human auditory neuroimaging of intensity and loudness. *Hear. Res.* 307 65–73. 10.1016/j.heares.2013.08.005 23973563

[B45] Woods D. L. Stecker G. C. Rinne T. Herron T. J. Cate A. D. Yund E. W. (2009). Functional maps of human auditory cortex: effects of acoustic features and attention. *PLOS ONE* 4:e5183. 10.1371/journal.pone.0005183 19365552 PMC2664477

[B46] Zuiderbaan W. Harvey B. M. Dumoulin S. O. (2012). Modeling center-surround configurations in population receptive fields using fMRI. *J. Vis.* 12:10. 10.1167/12.3.10 22408041

